# Deconvolution of Serum Cortisol Levels by Using Compressed Sensing

**DOI:** 10.1371/journal.pone.0085204

**Published:** 2014-01-28

**Authors:** Rose T. Faghih, Munther A. Dahleh, Gail K. Adler, Elizabeth B. Klerman, Emery N. Brown

**Affiliations:** 1 Department of Electrical Engineering and Computer Science, Massachusetts Institute of Technology, Cambridge, Massachusetts, United States of America; 2 Laboratory for Information and Decision Systems, Massachusetts Institute of Technology, Cambridge, Massachusetts, United States of America; 3 Department of Anesthesia, Critical Care and Pain Medicine, Massachusetts General Hospital, Boston, Massachusetts, United States of America; 4 Institute for Medical Engineering and Science, Massachusetts Institute of Technology, Cambridge, Massachusetts, United States of America; 5 Harvard Medical School, Boston, Massachusetts, United States of America; 6 Division of Endocrinology, Diabetes and Hypertension, Brigham and Women's Hospital, Boston, Massachusetts, United States of America; 7 Division of Sleep Medicine, Brigham and Women's Hospital, Boston, Massachusetts, United States of America; 8 Department of Brain and Cognitive Sciences, Massachusetts Institute of Technology, Cambridge, Massachusetts, United States of America; John Hopkins University School of Medicine, United States of America

## Abstract

The pulsatile release of cortisol from the adrenal glands is controlled by a hierarchical system that involves corticotropin releasing hormone (CRH) from the hypothalamus, adrenocorticotropin hormone (ACTH) from the pituitary, and cortisol from the adrenal glands. Determining the number, timing, and amplitude of the cortisol secretory events and recovering the infusion and clearance rates from serial measurements of serum cortisol levels is a challenging problem. Despite many years of work on this problem, a complete satisfactory solution has been elusive. We formulate this question as a non-convex optimization problem, and solve it using a coordinate descent algorithm that has a principled combination of (i) compressed sensing for recovering the amplitude and timing of the secretory events, and (ii) generalized cross validation for choosing the regularization parameter. Using only the observed serum cortisol levels, we model cortisol secretion from the adrenal glands using a second-order linear differential equation with pulsatile inputs that represent cortisol pulses released in response to pulses of ACTH. Using our algorithm and the assumption that the number of pulses is between 15 to 22 pulses over 24 hours, we successfully deconvolve both simulated datasets and actual 24-hr serum cortisol datasets sampled every 10 minutes from 10 healthy women. Assuming a one-minute resolution for the secretory events, we obtain physiologically plausible timings and amplitudes of each cortisol secretory event with *R* ^2^ above 0.92. Identification of the amplitude and timing of pulsatile hormone release allows (i) quantifying of normal and abnormal secretion patterns towards the goal of understanding pathological neuroendocrine states, and (ii) potentially designing optimal approaches for treating hormonal disorders.

## Introduction

Since many endocrine hormones such as cortisol are released in pulses instead of having slowly varying concentrations, understanding normal and abnormal endocrine functioning requires detection and quantification of both timing and amplitude of hormone pulses [1]. Determining the number, timing, and amplitude of hormone pulses is a challenging problem. For hormones that are released under control of the hypothalamus and anterior pituitary, release is regulated hierarchically; hypothalamic hormone release induces pituitary hormone release [2]. Then, either the pituitary hormone induces the release of another hormone from an endocrine gland (e.g. cortisol from the adrenal glands or thyroid from the thyroid gland) or the hormone of interest is directly released from the pituitary (e.g. growth hormone). These hormones are absorbed from the blood stream, and implement regulatory functions throughout the body. Some hormones exert negative feedback on release of their hypothalamic and pituitary regulatory hormones, and consequently their further release [2].

Current data analysis methods for pulsatile hormone secretion either assume that the timing of the impulses belongs to a certain class of stochastic processes (e.g. birth-death process) [3] or use pulse detection algorithms [4]. The problem of recovering the model parameters as well as the number, timing, and amplitude of hormone pulses from a limited number of observations is ill-posed (i.e., there could be multiple solutions). However, by taking advantage of the sparse nature of hormone pulses and adding more constraints, the problem becomes more tractable. Veldhuis et al. [5] and Keenan et al. [6] have recently reviewed various methods used for analyzing pulsatile hormone secretion. One method used for analyzing hormones is to assume point process models for the secretory events and employ methods such as the Markov Chain Monte Carlo (MCMC) algorithm [3]. For example, Johnson et al. find hormone pulses by embedding a birth-death process in an MCMC algorithm [3]. Keenan et al. propose an algorithm that uses a Bayesian approach to identify the pulses, and the secretion and clearance rates [6]. These methods assume that the occurrence of the secretory events is stochastic. In a recent work, Vidal et al. [4] use a pulse detection algorithm and remove peaks whose heights are small compared to the other detected pulses or some threshold. This pulse detection algorithm is implemented on luteinizing hormone (LH) data from ewes. In the LH data from ewes, one pulse decays significantly before the next pulse occurs. GH time series patterns also consist of hormone pulses that decay significantly before the next pulse occurs. However, one cortisol pulse can occur before the previous one has significantly decayed to near zero. Therefore, the extraction of the pattern of pulses may be less clear in cortisol than in LH and GH data, and analyzing cortisol data is more challenging.

As a first step in the study of novel methods of quantifying pulsatile activity of hormones, we investigate cortisol secretion in the hypothalamic-pituitary-adrenal (HPA) axis. Glucocorticoids, including cortisol, are crucial in neurogenesis, glucose homeostasis, metabolism, stress response, cognition, and response to inflammation [7]. Diseases that are linked to abnormalities in the HPA axis include diabetes, visceral obesity and osteoporosis, life-threatening adrenal crises and disturbed memory formation [8], [9]. Cortisol secretion is initiated by the release of corticotropin releasing hormone (CRH) from the hypothalamus; CRH induces pulsatile release of adrenocorticotropin hormone (ACTH) from the anterior pituitary, and through their stimulation by ACTH, the adrenal glands produce cortisol. Cortisol exerts negative feedback effect on the release of CRH and ACTH, and consequently future cortisol release. Normal physiology includes variation of the cortisol level over a 24-hour period; these variations are due to the circadian modulation of the amplitude of the secretory events and ultradian modulation of the timing of the secretory events. Thus, there is a complex physiology required to produce the cortisol pulses and in particular this physiology depends critically on the release of CRH from the hypothalamus and pulsatile release of ACTH from the anterior pituitary. In this study, we are only concerned with the problem of estimating the pharmacokinetics properties of cortisol as well as the number, timing and amplitude of the cortisol pulses from the time-series of the serum cortisol levels. Over 24 hours, there are 15 to 22 secretory events [10], [11] with an average of 18 secretory events in both males and females [12]. Hence, since there are a small number of secretory events that are significant, these hormone pulses can be considered sparse and specific analytic techniques can be applied.

In this paper, we use the characteristic of the sparsity of hormone pulses (i.e., there are a small number of secretory events that are important) and recover the timing and amplitude of individual hormone pulses using compressed sensing techniques. Compressed sensing is a technique for perfect reconstruction of sparse and compressible signals using fewer measurements than required by the Shannon/Nyquist sampling theorem [13]. For compressible signals, where only a small number of coefficients are large (i.e., most coefficients are small or zero) and small coefficients can be discarded, the signal can be approximated by a sparse representation and recovered using optimization or greedy algorithms [13]. We describe a coordinate descent approach to recover cortisol secretory events and model parameters. To demonstrate the performance of the algorithm, we apply it to cortisol data. Although we know the sparsity range of the input, the number of pulses for each participant is an open question. In finding the number of pulses, there is a trade-off between capturing the residual error and the sparsity. We use generalized cross-validation to find the number of pulses such that there is a balance between the residual error and the sparsity. This algorithm potentially can be applied to other pulsatile endocrine hormones such as GH, thyroid hormone, LH, and testosterone.

## Methods

### Experiment

Blood from 10 healthy women collected every 10 minutes for 24 hours was assayed for cortisol in duplicate. The 24 hours began with 8 hours of scheduled sleep followed by 16 hours of wake. The participants were recruited via advertisements in local newspapers to serve as healthy controls for a study on women with fibromyalgia; participants with abnormal laboratory test results or current medical problems were excluded [14]. None of the participants had received glucocorticoids or estrogen/progesterone within the year or 4 months before the study, respectively [14]. For 3 consecutive nights, at the General Clinical Research Center of the Brigham and Womens Hospital, participants had 8 hours of scheduled sleep in the dark at their habitual sleep-wake times and three meals and two snacks; on day 4, all participants started a constant routine protocol that included continuous wake, constant posture, and small meals given hourly [14]. Blood drawing for hormones analyzed in this study was performed during the third night of sleep and during the first 16 hours of the constant routine. A detailed description of the experiment is in [14]; clinical characteristics of the participants are given in Table 1. The experimental protocol was designed to minimize the effects of ambulatory temperature, activity, eating, and stress on the participants. Therefore, this dataset can be used to quantify cortisol variations as a function of the time of the day, as controlled by the circadian and the ultradian patterns.

**Table 1:** Clinical Characteristics of the Participants.

AGE	BMI ( 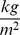 )	Systolic BP (mmHg)	Diastolic BP (mmHg)
28	20.8	100	70
41	23.6	120	80
41	22.5	130	70
24	20.7	108	78
23	27.4	108	68
26	25.2	108	68
23	29.3	110	74
37	29.6	138	80
44	29.9	135	82
42	22.9	108	62

BMI and BP refer to Body Mass Index and Blood Pressure, respectively. None of the participants had a current diagnosis of depression

### Ethics Statement

This project has been reviewed and approved by the Brigham and Women's Hospital Institutional Review Board (IRB). During the review of this project, the IRB specifically considered (i) the risks and anticipated benefits, if any, to subjects; (ii) the selection of subjects; (iii) the procedures for securing and documenting informed consent; (iv) the safety of subjects; and (v) the privacy of subjects and confidentiality of the data. All subjects provided written informed consent that was approved by the ethics committees.

### Model Formulation

We build a model based on the stochastic differential equation model of diurnal cortisol patterns [10]. This model is based on the first-order kinetics for cortisol synthesis in the adrenal glands, cortisol infusion to the blood, and cortisol clearance by the liver, while considering a doubly stochastic pulsatile input in the adrenal glands that has Gaussian amplitudes and gamma distributed interarrival times [10]. This input can be considered as an abstraction of hormone pulses and marks the timing and amplitude of the secretory events leading to cortisol secretion. We assume that there are between 15 and 22 secretory events over a 24-hour period that result in the observed cortisol profile [10], [11], [15], [16]. This model is represented as follows:
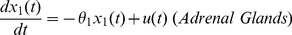
(1)

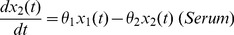
(2)where *x*_1_ is the cortisol concentration in the adrenal glands and *x*_2_ is the serum cortisol concentration. *θ*_1_ and *θ*_2_, respectively, represent the infusion rate of cortisol from the adrenal glands into the blood and the clearance rate of cortisol by the liver. 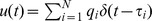 is an abstraction of the hormone pulses that result in cortisol secretion where *q_i_* represents the amount of the hormone pulse initiated at time *τ_i_* (*q_i_* is zero if a hormone pulse did not occur at time *τ_i_*), and we assume that impulses occur at integer minute values. *N* corresponds to the length of the input (*N* = 1440).

Blood was collected, beginning at *y*_0_ and then, with a sampling interval of 10 minutes, for *M* samples (*M* = 144). All samples were assayed for cortisol. Let 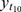, 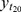,…, 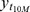


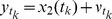
(3)where 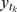 and 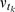 represent the observed serum cortisol level and the measurement error, respectively, and missing data points can be interpolated. For each participant, blood samples were assayed in duplicate; hence, for each participant, we could obtain the standard deviation of noise and model the corresponding measurement error. A Gaussian density is a reasonably good approximation of the probability density of the immunoassay error [10], and, by using a least squares approach in our estimation algorithm, we model the noise as a Gaussian random variable. Using the serum cortisol level (*x*_2_) with a sampling interval of 10 minutes, we would like to estimate *θ*_1_ and *θ*_2_, and obtain the number of pulses, their timing, and their amplitude.

Considering the known physiology of 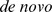 cortisol synthesis (i.e., no cortisol is stored in the adrenal glands) [10], we assume that the initial condition of the cortisol level in the adrenal glands is zero (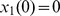) [3], and solve for 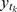. Considering that we have discrete data points sampled every 10 minutes, assuming that the input occurs at integer minutes, and 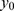 is the initial condition of the serum cortisol concentration, every observed data point 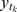 can be represented as follows:
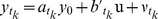
(4)where 
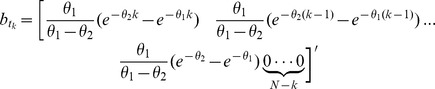
,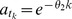, and u represents the entire input over 24 hours (elements of u take values 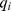 for 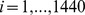).

Let 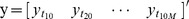, 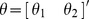, 
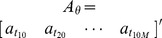
, 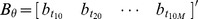, and 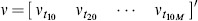.

We can represent this system as:
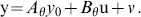
(5)


### Estimation

In modeling cortisol secretion over 24 hours, results from previous studies suggest that there are 15 to 22 secretory events [10], [11], [15], [16], and on average, there are 18 secretory events [12]. Hence, we assume u contains 15 to 22 nonzero elements out of 1440 possibilities and all these nonzero elements are nonnegative (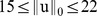, 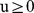). To estimate the model parameters, we follow [10] and assume that the infusion rate of cortisol from the adrenal glands to the circulation is at least four times the clearance rate of cortisol by the liver (i.e., 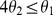). Because the hormone infusion and clearance rates cannot be negative, we further assume in the optimization algorithm that 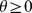. We can formulate this problem as an optimization problem:
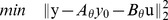
(6)

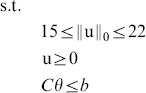
where 
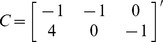
, 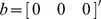.

This optimization problem is generally considered as NP-hard. It is possible to use an 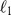-norm relaxation and solve this problem using different computational strategies such as the basis pursuit, greedy algorithms, iterative-thresholding algorithms, or the FOCUSS algorithm and its extensions [17]. We solve this problem with an extension of the FOCUSS algorithm by first casting this optimization problem as:
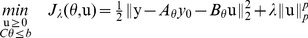
(7)where the 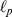-norm is an approximation to the 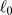-norm (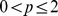) and *λ* is chosen such that the sparsity of u is between 15 to 22. Then, by using a coordinate descent approach, this optimization problem can be solved iteratively through the following steps (for 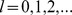) until convergence is achieved:

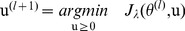
(8)

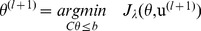
(9)

The optimization problem in (8) now can be solved using the FOCUSS algorithm, which uses a re-weighted norm minimization approach. The solution at each iteration is found by minimizing the 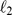-norm and the iteration refines the initial estimate to the final localized energy solution [18]. In the FOCUSS algorithm, assuming that a gradient factorization exists, the stationary points of (8) satisfy 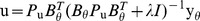 [18], where 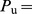
diag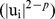, and 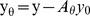. By iteratively updating *λ* and u until convergence, we can solve for the sparse vector u. In the optimization problem in (8), *λ* balances between the sparsity of u and the residual error 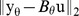. The sparsity of u increases with *λ*.

A version of the FOCUSS algorithm called FOCUSS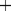 proposed by Murray [19] allows for solving for u such that the maximum sparsity of u is *n* (*n* = 22 for our current problem) and u is nonnegative. This algorithm uses a heuristic approach for updating *λ*, which tunes the trade-off between the sparsity and the residual error by increasing *λ* to a maximum regularization 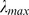 as the residual error decreases. FOCUSS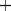 works as follows:

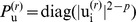

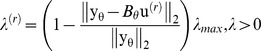

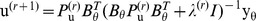

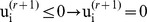

5. After more than half of the selected number of iterations, if 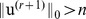, select the largest *n* elements of 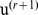 and set the rest to zero.
6. Iterate

FOCUSS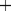 usually converges in 10 to 50 iterations [19]. Although an estimate of the unknown quantities can be obtained after iteratively solving for u and *θ* (8–9) using FOCUSS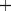, *θ* and u should be updated by finding an optimal choice of *λ* such that enough noise is filtered out and the estimated u is not capturing residual error by finding a less sparse solution. We use the Generalized Cross-Validation (GCV) technique [20] for estimating the regularization parameter such that there is a balance in filtering out the noise and the sparsity of u. The GCV function is defined as:
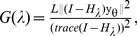
(10)where *L* is the number of data points, and *H_λ_* is the influence matrix. For the FOCUSS algorithm, 
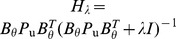
. Zhaoshui et al. [17] employ the GCV technique for estimating the regularization parameter for the FOCUSS algorithm through singular value decomposition:
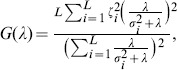
(11)where 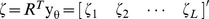 and 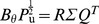 with 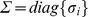; *R* and *Q* are unitary matrices and 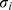
's are the singular values of 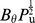. [17]. Zhaoshui et al. [17] propose minimizing *G*(*λ*) such that *λ* is bounded between some minimum and maximum values (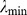 and 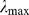) using the MATLAB function 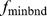 (MATLAB R2011b), which is an implementation of the golden section (GS) search. Although the GS search only finds a local extremum, considering that *G*(*λ*) is unimodal, the GS search always finds the desired solution given a large range for *λ* [17]. We used a range of zero to 10 for *λ*.

Considering the ill-posedness of deconvolution problems, small variations in the data can result in large changes in the solution, and a balanced choice of regularization is required to filter out the effect of noise. Tikhonov regularization, truncated singular value decomposition, and the method of L-curve are well-known methods used when dealing with such problems [21]; among these methods, the L-curve method appears to be the most commonly used. Automatically searching for the minimum of the GCV function is easier than finding the corner of the L-curve as the L-curve method is computationally expensive, requiring computation of the solution for several samples of the parameter [17]. Moreover, Zhaoshui et al. point out that GCV is usually more accurate in estimating the regularization parameter than the L-curve method [17]. In the GCV technique, the optimal choice of regularization minimizes the predictive mean-squared error. Hence, one can update u by modifying FOCUSS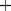 using the GCV method. For 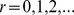 GCV - FOCUSS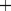 works as follows:

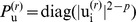

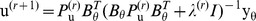

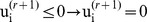

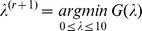

5. Iterate until convergence

By combining the GCV method with FOCUSS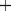, one can find an optimal choice of *λ* at each iteration such that enough noise is filtered out when solving for u, and iterate between solving (8) and (9) until convergence is achieved. The following is the algorithm that we propose for deconvolution of cortisol data:

1. Initialize 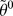 by sampling a uniform random variable 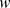 on 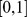, and let 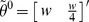 and 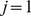

2. Set 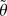 equal to 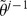; using FOCUSS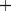, solve for 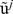 by initializing the optimization problem in (8) at a vector of all ones
3. Set 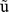 equal to 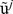; using the interior point method, solve for 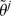 by initializing the optimization problem in (9) at 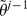

4. Iterate between steps 2–3 for 30 iterations
5. Initialize 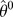 and 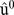 by setting them equal to the 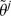 and 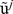 that minimize 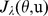 in (7), and let *i* = 1
6. Set 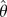 equal to 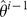; using GCV −00 pt FOCUSS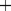, solve for 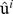 by initializing the optimization problem in (8) at 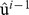

7. Set 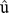 equal to 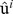; using the interior point method, solve for 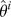 by initializing the optimization problem in (9) at 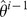

8. Iterate between steps 6–7 until convergence
9. Repeat steps 1–8 for various initializations
10. Set the estimated model parameters 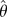 and input 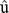 equal to the values that minimize 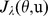 in (7). Since this optimization problem is non-convex, there are multiple local minima, and a reasonable procedure to choose among the local minima is to select the one with the best goodness of fit.

Step 1 initializes the algorithm randomly. Steps 2–4 use the random initialization to find a good initialization for the unknowns while using FOCUSS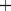 for sparse recovery and interior point method for finding the model parameters. Step 5 finds a good initial condition by comparing the estimates obtained in Steps 2–4, and selecting the *θ* and u values that minimize the cost function. The values found at Step 5 are used for initializing the main algorithm. Steps 6–8 use a coordinate descent approach to estimate the unknowns until convergence is achieved. Sparse recovery is achieved using GCV − FOCUSS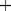 which uses generalized cross-validation for finding the regularization parameter; GCV − FOCUSS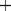 selects the regularization parameter such that there is a balance between capturing the sparsity and the noise, and finds different sparsity levels for different individuals. Step 9 repeats the initialization and estimation steps for various initializations. Step 10 selects the *θ* and u values that minimize the cost function.

The main idea behind the algorithm is to solve the non-convex problem in (7) using a coordinate descent approach to converge to a local minimum for different initializations, and choose the local minimum that minimizes the problem in (7). Convergence properties of coordinate descent algorithms are well-studied and a discussion can be found in [22].

We have implemented the algorithm by assuming that hormone pulses occur at integer minutes. We ran the proposed algorithm using 10 random initializations for each dataset. According to [23], the FOCUSS algorithm converges faster for 0<*p*<1 compared to 1<*p*<2; however, it should be noted that for 0<*p*<1, the optimization problem is not convex and if *p* is too small (e.g., *p* = 0.1), it is possible to stagnate into a local minimum; hence, they suggest selecting a value slightly smaller than 1 but not too small [23]. When running GCV − FOCUSS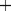, we let *p* = 0.5 to solve for u. Data analysis, estimation, and simulations were performed in MATLAB R2011b.

**Figure 1:**
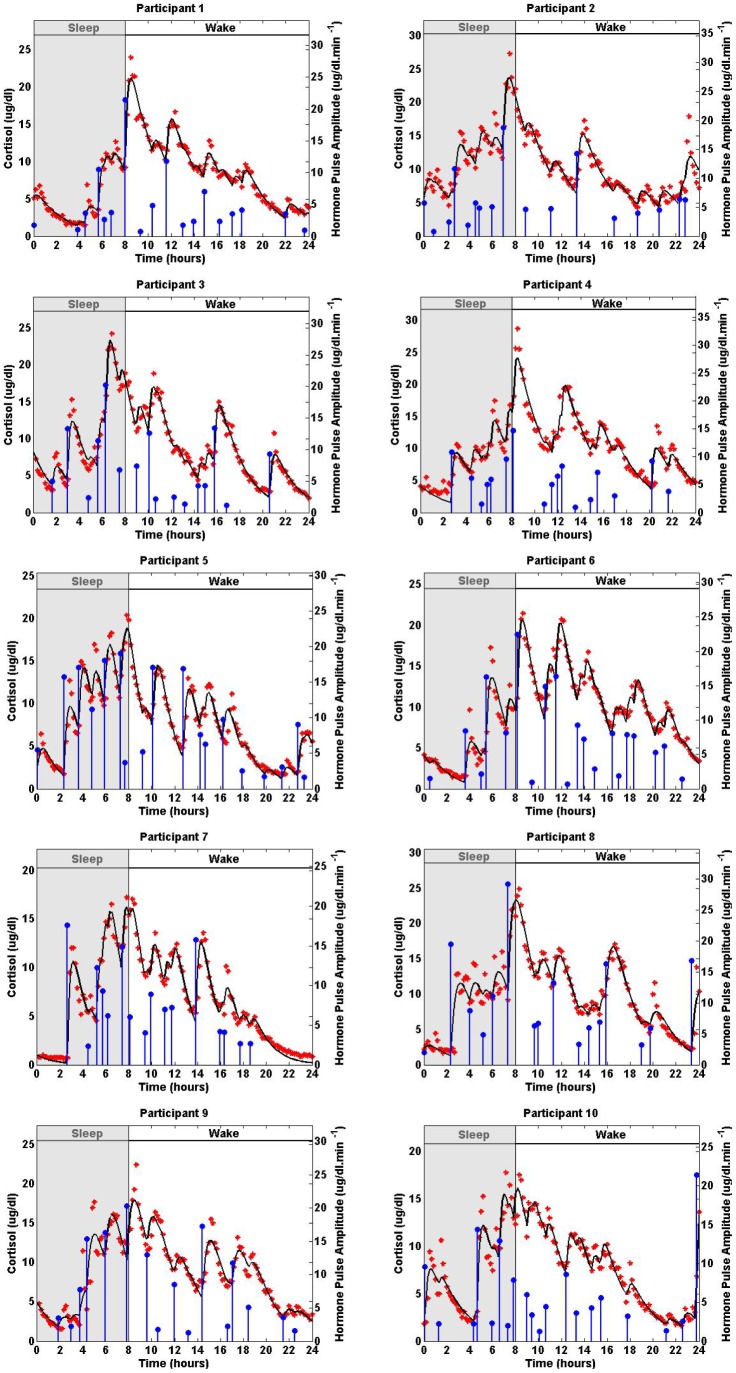
Estimated Deconvolution of the Experimental Twenty-Four-Hour Cortisol Levels in 10 Women. Each panel shows the measured 24-hour cortisol time series (red stars), the estimated cortisol levels (black curve), the estimated pulse timing and amplitudes (blue vertical lines with dots) for one of the participants. The estimated model parameters are given in Table 2.

Using the cortisol secretion model (1–2), we simulated ten 24-hour cortisol datasets using parameters 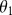 and 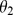 in Table 2 and impulse trains in Figure 1. Then, using the observation model (3), we added zero mean Gaussian noise with a standard deviation of 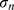 to the simulated cortisol levels; 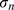 in Table 3 models the immunoassay error for each participant and was obtained using blood samples assayed in duplicate.

**Table 2:** The Estimated Model Parameters and the Squares of the Multiple Correlation Coefficients (*R* ^2^) for the Fits of the Experimental Cortisol Time Series.

Participant	*θ*_1_(min^−1^)	*θ*_2_(min^−1^)	N	*R* ^2^
1	0.0739	0.0067	18	0.97
2	0.0762	0.0057	17	0.93
3	0.0921	0.0082	16	0.96
4	0.1248	0.0061	17	0.93
5	0.0585	0.0122	18	0.95
6	0.0726	0.0095	20	0.96
7	0.0799	0.0107	16	0.97
8	0.0365	0.0091	16	0.93
9	0.0361	0.0090	16	0.92
10	0.0864	0.0073	20	0.94
Median	0.0751	0.0086	17	0.95

The parameters 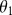 and 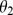 are, respectively, the estimated infusion rate of cortisol into the circulation from the adrenal glands and the estimated clearance rate of cortisol by the liver. *N* is the estimated number of hormone pulses and *R* ^2^ is the square of the multiple correlation coefficient.

**Table 3:** The Estimated Model Parameters and the Squares of the Multiple Correlation Coefficients (*R* ^2^) for the Fits of the Simulated Cortisol Time Series.

Participant	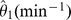	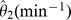	N	*R* ^2^	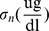	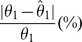	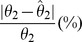
1	0.0628	0.0068	16	0.99	0.38	15.02%	1.49%
2	0.0569	0.0056	15	0.97	0.75	25.33%	1.75%
3	0.0747	0.0078	15	0.98	0.67	18.89%	4.88%
4	0.0918	0.0071	16	0.94	1.44	26.44%	16.39%
5	0.0492	0.0123	18	0.99	0.52	15.90%	0.82%
6	0.0490	0.0122	19	0.99	0.29	32.51%	28.42%
7	0.0770	0.0098	16	0.97	0.98	3.63%	8.41%
8	0.0375	0.0094	18	0.99	0.33	2.74%	3.30%
9	0.0365	0.0091	16	0.99	0.35	1.11%	1.11%
10	0.0654	0.0076	19	0.99	0.31	24.30%	4.11%
Median	0.0599	0.0084	16	0.99	0.45	17.39%	3.70%

The parameters 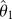 and 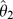 are, respectively, the estimated infusion rate of cortisol into the circulation from the adrenal glands and the estimated clearance rate of cortisol by the liver. *N* is the estimated number of hormone pulses and *R* ^2^ is the square of the multiple correlation coefficient. 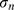 is the standard deviation of the zero mean Gaussian noise added to each simulated data point. For each participant, blood samples were assayed in duplicate, and the corresponding standard deviation of noise was obtained. The parameters 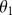 and 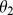 are, respectively, the infusion rate of cortisol into the circulation from the adrenal glands and the clearance rate of cortisol by the liver used in simulating each dataset. The values of 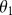 and 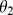 are given in Table 2.

## Results

Figure 1 shows experimental data from each participant. For most participants, the cortisol level is low at the beginning of the scheduled sleep; the cortisol level increases rapidly around the wake time and gradually decreases throughout the day.

**Figure 2:**
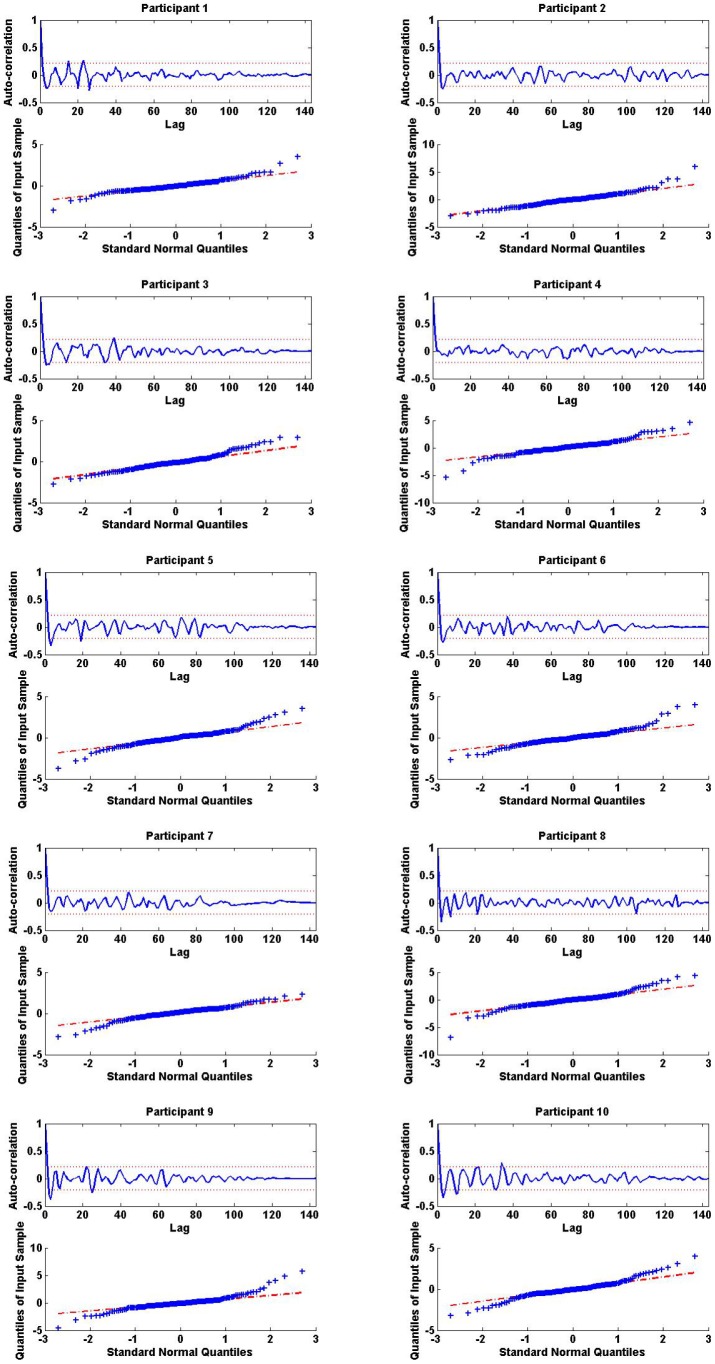
White Gaussian Structure in the Model Residuals of 10 Women. In each panel, (i) the top sub-panel displays the autocorrelation function of the model residuals in one of the 10 participants; the graph shows that the model captures the dynamics and that residuals are white; (ii) the bottom sub-panel displays the quantile-quantile plot of the model residuals for that participant; the graph shows that the residuals are Gaussian.

Figure 1 shows the estimated amplitude and timing of hormone pulses, experimental cortisol data, and model-predicted cortisol estimates for each participant. There are variations in the timing and amplitudes of the detected hormone pulses. The circadian amplitudes of the recovered pulses demonstrate the known circadian variation of cortisol time series [10]; for most participants, the recovered pulses are small at the beginning of the scheduled sleep, and there is a large pulse towards the end of the sleep period or beginning of the wake period. There are multiple small and medium sized pulses during the wake period. The number of detected pulses for all participants are within their corresponding physiologically plausible ranges [10], [11], [15], [16] with a square of the multiple correlation coefficient (*R* ^2^) above 0.92 (Table 2). The *R* ^2^ is a statistical measure of the goodness of fit obtained by model-predicted estimates of data; it measures the fraction of the sample variance of data that is predicted by the model: *R* ^2^ values close to 1.0 suggest that the model is good at estimating the data. Figure 2 shows the autocorrelation function and the quantile-quantile plots of the model residuals for the 10 participants suggesting that the model captures the dynamics, and that the residuals have a Gaussian structure and are white.

**Figure 3:**
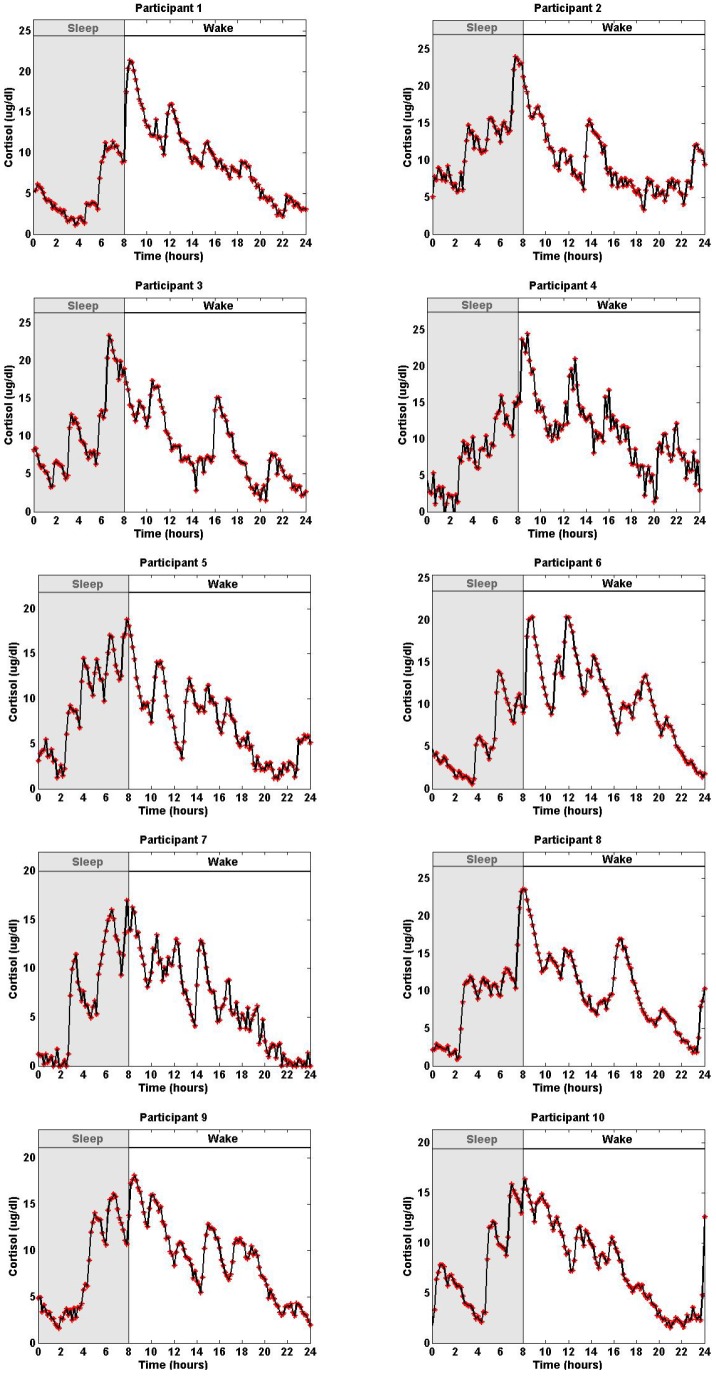
Simulated Twenty-Four-Hour Cortisol Levels with Measurement Errors Corresponding to Datasets from 10 Women. Each panel displays the simulated serum cortisol levels based on pulse patterns in Figure 1 and estimated model parameters 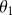 and 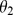 in Table 2 in one of the 10 participants, assuming a zero mean Gaussian measurement error with standard deviation 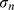 in Table 3. In all simulations the initial conditions are 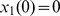, 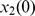 equals the initial cortisol level of the corresponding participant, and the cortisol levels are recorded every 10 minutes.

We simulated 10 datasets, each corresponding to one of the 10 experimental datasets (Figure 3). These datasets were simulated by using the recovered pulses of the 10 experimental datasets shown in Figure 1 as well as the estimated model parameters for each of the corresponding datasets as shown in Table 2. Then, we added zero mean Gaussian noise with standard deviations shown in Table 3 to the simulated data. These 10 simulated datasets were sampled every 10 minutes.

Table 3 shows the estimated model parameters, number of pulses, the square of the multiple correlation coefficient (*R* ^2^), the percentage error in estimating the model parameters, and the standard deviation of zero mean Gaussian noise used in simulating the 10 datasets. These simulations were performed by adding various values of zero mean Gaussian noise with standard deviations 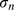 ranging from 0.29 to 1.44; the level of noise was based on the signal-to-noise ratio for data collection for each of the participants. Errors in estimating 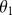 and 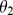 range from small values (1.11% and 0.82%) to high values (32.51% and 28.42%), respectively. The maximum error in finding the number of pulses is two. There is zero error in finding the number of pulses for participant 7 where the maximum error in the detected support is 14 minutes. The overall performance of the estimation for the simulated dataset is best for the data that corresponds to participant 7 with 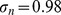, and 3.63% and 8.41% error in estimating 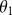 and 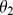, respectively. These estimates were obtained by 10 random initializations, and considering that the optimization problem solved for this estimation is non-convex, there are multiple local minima and the estimation can be improved by using more initializations. Since the noise added to the simulated data is comparable in amplitude to small pulses of cortisol, the pulsatile patterns of the simulated cortisol time series differ from their corresponding experimental time series. This explains the higher error in the estimated model parameters 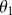 and 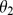, and the error in the estimated timing and amplitudes of pulses. This choice of noise is based on the immunoassay error for each experimental time series, so that the simulations are based on multiple metrics of the experimental data. The algorithm performs better for lower levels of noise that do not significantly affect the pulsatile patterns of the simulated time series.

**Figure 4:**
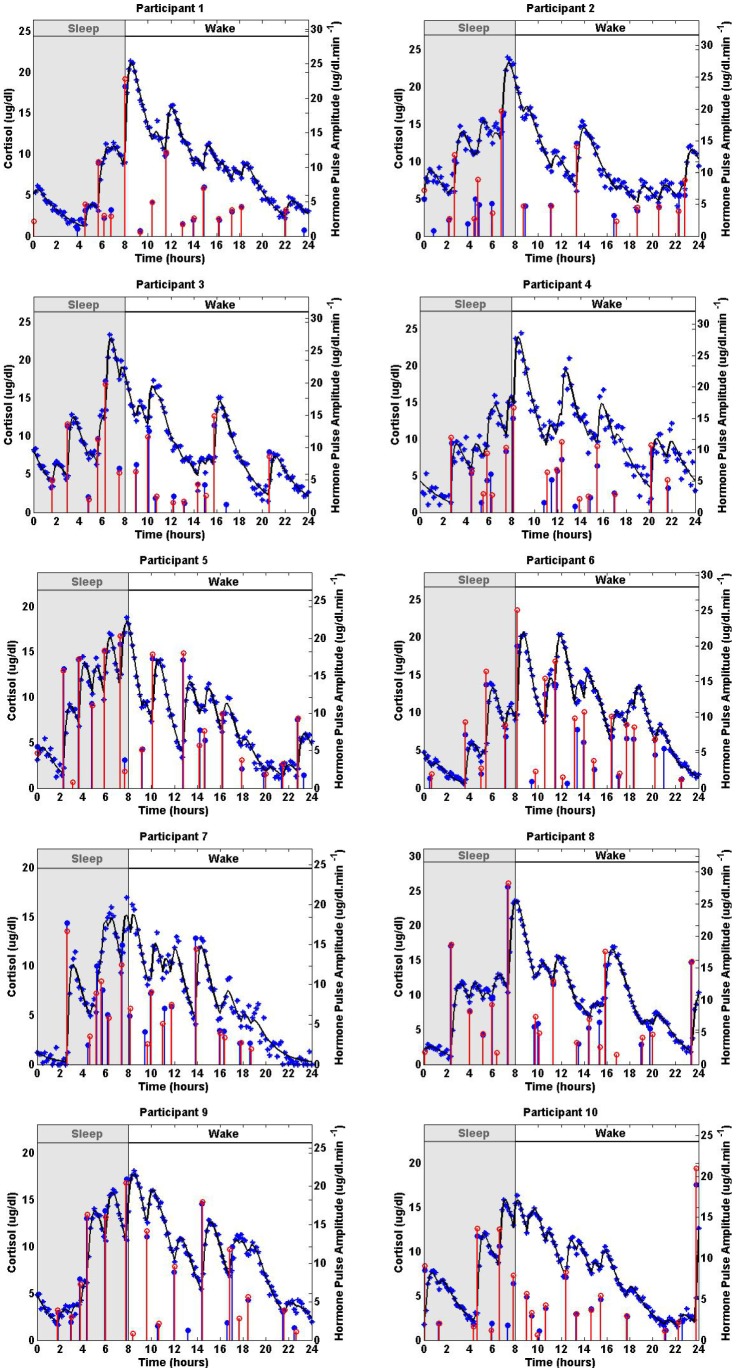
Estimated Deconvolution of Simulated Twenty-Four-Hour Cortisol Levels with Different Measurement Errors Corresponding to Datasets from 10 Women. Each panel shows the simulated 24-hour cortisol time series (blue stars), the estimated cortisol levels (black curve), the simulated pulse timing and amplitudes (blue vertical lines with dots) and the estimated pulse timing and amplitudes (red vertical lines with empty circles) for one of the simulated datasets that each correspond to a participant. The estimated parameters are given in Table 3.

Figure 4 shows the actual sparse input, the recovered input, the simulated cortisol data, and the estimated cortisol levels. Table 4 shows the maximum error in the detected timing of pulses, the number of small pulses that are not detected, and the number of extra pulses that are detected for each simulated dataset. The recovered and the actual inputs are in good agreement for a variety of noise levels when detecting significant pulses; however, a few of the small pulses cannot be detected for some cases or in some cases noise is captured as a small pulse. The maximum error in detecting the support is 26 minutes.

**Table 4:** The Error in Estimated Pulses of the Simulated Cortisol Time Series.

Participant	M*_e_* (min)	*N_u_*	*N_d_*
1	4	2	0
2	14	2	0
3	6	1	0
4	26	1	0
5	10	1	1
6	22	1	0
7	14	0	0
8	22	0	2
9	13	2	2
10	22	1	0

M*_e_* is the maximum error in the detected timing of pulses. *N_u_* and *N_d_* are, respectively, the number of pulses that are not detected and the number of extra pulses that are detected for each simulated dataset.

## Discussion

Understanding the cortisol secretion process and modeling the underlying system is a challenging problem for several reasons. (I) Due to the simultaneous release and clearance of hormones and the unknown timing and amplitudes of the secretory events, identifying the pulsatile input to the system and the infusion and clearance rates is challenging. (II) Due to data collection difficulties and cost, the sampling interval is usually relatively large (10–60 minutes) compared to the expected inter-pulse intervals as well as secretion and clearance rates of cortisol. This low resolution of the data makes identifying the delays in the system and potential consecutive pulses that occur over one sampling interval challenging. (III) Cortisol secretion differs in sleep and wake states and at different circadian times; therefore, the model parameters might be time-varying. (IV) There is inter-individual variation, even among healthy individuals. (V) The properties of noise in the system are not known.

In this paper, we modeled secretory events that result in cortisol time series, and proposed a coordinate descent approach to estimate the model parameters and recover the sparse time-varying secretory input. Considering the sparsity of the input, we recovered the impulses using compressed sensing. While a range for the sparsity of the hormone pulses is known, the exact number of pulses varies from one individual to another and is unknown. To recover the accurate number of hormone pulses, the regularized problem should be solved such that there is a balance between capturing the sparsity and the residual error. We used generalized cross-validation for choosing the regularization parameter and finding the number of pulses for each individual. The algorithm described in this paper provides a general framework that can also be implemented on other hormones. For the case of cortisol data, the high *R* ^2^ values (found to be greater than 0.92 for all 10 participants) suggest that our proposed algorithm can successfully uncover physiologically plausible hormone pulse information underlying cortisol secretion. There are variations in the timing and amplitudes of cortisol secretory events. The amplitude variations throughout the day occur as a result of the circadian rhythm underlying cortisol release; the variations in the timing of impulses reflect the ultradian rhythm underlying cortisol release. Our algorithm makes it possible to capture the circadian and ultradian features of hormone pulses as well as the parameters underlying the first-order kinetics of cortisol release. Furthermore, our algorithm recovered the impulse train input from simulated data with various noise levels and detected the significant pulses; however, depending on the dataset, it misses one or two of the insignificant pulses or captures noise as one or two small pluses. Our approach can be applied to GH, thyroid hormone, and gonadal hormones in a similar fashion. Future directions for this research include modeling simultaneous measurements of ACTH and cortisol data. In addition, as we postulate that the human body controls hormone secretion by solving an optimization problem, we can use these methods to begin to understand the underlying physiology. Correlation analysis of the residuals suggests that the model captures the dynamics, and residuals are Gaussian and white.

Many data analysis methods for modeling hormone pulsatility either assume the timing of the impulses belongs to a certain class of stochastic processes or use pulse detection algorithms [3], [4]. These procedures work well when the pulses are readily identifiable and are more challenging when the pulses are more difficult to discern by visual inspection as in the case of cortisol. Johnson et al. [3] and Vidal et al. [4], respectively, analyze LH data using a birth-death process in an MCMC algorithm and a pulse detection algorithm. While our algorithm also can be applied to LH, we analyzed cortisol data, in which the timing of the hormone pulses is not as clearly defined as in LH data. In summary, our proposed algorithm works well even when the pulses are not easily identifiable while still being applicable to cases in which pulses are identifiable by visual inspection. Furthermore, our algorithm does not require assumptions about the inter-arrival times of the pulses, and timings of pulses can be recovered for different classes of distributions of inter-arrival times.

Although our proposed algorithm runs on average in less than half an hour, it can be accelerated. In the data that we analyzed, the likelihood of two pulses occurring during small intervals is low because the experiment was conduced when the participants were under relatively low stress, were fed regularly, had low constant activity levels, and the ambient temperature was held constant. We have not tested our algorithm on data from settings in which the likelihood of two impulses occurring in short intervals is high due to external factors such as stress. Moreover, in our analysis, we make the assumption that the pulses occur at any minute; hence, the detected pulses could have happened within a minute before or after the pulse was detected. Using an approach similar to the one proposed here, with an appropriate number of data points, one could bin the data assuming the pulses occur at any second, or even millisecond and detect the pulses with a higher resolution.
